# Acute Events and Fatal Outcomes Following Exposure to Volcanic Gases (SO_2_, CO_2_, H_2_S): A Systematic Review

**DOI:** 10.3390/medsci14050531

**Published:** 2026-08-31

**Authors:** Cristina Genovese, Giovanni Paolo Piccolo, Alessandro Gattuso, Antonio Mistretta, Francesco Leonforte, Daniela Lo Giudice, Daniele Maisano, Raffaele Squeri, Caterina Elisabetta Rizzo

**Affiliations:** 1 Department of Biomedical Sciences, Dental Sciences and Morpho-Functional Imaging, University of Messina, 98100 Messina, Italy; 2 Department of Biomedical Sciences, Dental Sciences and Morpho-Functional Imaging, School of Medicine, University of Messina, 98100 Messina, Italy; 3 Palermo and Milazzo Operational Unit, National Institute of Geophysics and Volcanology (INGV), 98057 Milazzo, Italy; 4 Section of Hygiene and Preventive Medicine, Department of Medical and Surgical Sciences and Advanced Technologies “G.F. Ingrassia”, University of Catania, 95100 Catania, Italy; anmist@unict.it; 5 Department of Integrated Hygiene, Organizational, and Service Activities (Structural Department), Health Management, University Hospital Polyclinic “G. Rodolico-San Marco”, 95100 Catania, Italy; francesco.leonforte@policlinico.unict.it; 6 Department of Prevention, Local Health Authority of Enna, 94100 Enna, Italy

**Keywords:** volcanic gases, acute intoxication, public health, systematic review

## Abstract

Background: Volcanic gas emissions represent an important but frequently underestimated non-eruptive natural hazard capable of causing severe acute intoxication and sudden death, even in the absence of volcanic eruptions. Although numerous reviews have investigated the chronic health effects of volcanic emissions, evidence regarding acute exposure events remains fragmented. This systematic review aimed to synthesize the available evidence on acute intoxication events and fatalities associated with exposure to sulfur dioxide (SO_2_), carbon dioxide (CO_2_), and hydrogen sulfide (H_2_S) of volcanic origin. Materials and Methods: A systematic literature search was conducted according to the PRISMA 2020 guidelines across six electronic databases (PubMed/MEDLINE, Scopus, Web of Science, Embase, Google Scholar, and WHO IRIS). Results: Six eligible studies describing fatal and non-fatal acute exposure events from five countries (Italy, France, the United States, Cameroon, and the Democratic Republic of Congo) were included. Carbon dioxide and hydrogen sulfide were the gases most frequently associated with fatal outcomes, predominantly occurring in confined spaces, geothermal areas, fumaroles, topographic depressions, or environments where adverse meteorological conditions promoted gas accumulation. The most common clinical manifestations included sudden loss of consciousness, pulmonary edema, respiratory failure, and asphyxial death. The largest documented event, the 1986 Lake Nyos disaster, resulted in more than 1700 fatalities following a massive limnic release of carbon dioxide. Conclusions: Across the included studies, recurring environmental determinants, exposure pathways, and toxicological mechanisms highlighted the critical role of environmental accumulation in shaping exposure risk. Despite the limited number of published cases, the available evidence demonstrates that acute volcanic gas exposure constitutes a significant yet underrecognized public health hazard. Strengthening environmental monitoring, risk communication, visitor safety measures, and interdisciplinary surveillance systems may substantially reduce preventable morbidity and mortality in volcanically active regions.

## 1. Introduction

Volcanically active environments represent complex ecological and geophysical systems characterized by multiple natural hazards that may threaten human health [1]. While volcanic eruptions are typically regarded as the most dramatic manifestation of volcanic activity, non-eruptive hazards such as persistent volcanic degassing represent a significant but often underestimated source of risk [2]. Volcanic gas emissions may occur continuously, even in the absence of eruptive activity, and can lead to hazardous environmental conditions capable of producing acute toxic effects and fatal outcomes [3].

Among volcanic emissions, sulfur dioxide (SO_2_), carbon dioxide (CO_2_), and hydrogen sulfide (H_2_S) are the gases most frequently implicated in acute exposure events [4]. These gases exhibit physicochemical properties that enhance their hazard potential [5]. Their density relative to air favors accumulation in topographic depressions, enclosed spaces, geothermal fields, fumaroles, and poorly ventilated environments [6]. Consequently, localized gas concentrations may reach levels incompatible with life.

Hydrogen sulfide presents an additional risk due to olfactory fatigue, whereby high concentrations rapidly impair the sense of smell, eliminating the characteristic warning odor [7]. Carbon dioxide, being colorless and odorless, cannot be detected without monitoring devices. These characteristics contribute to sudden exposure events with rapid onset of toxic effects [8].

Existing literature has extensively documented the chronic respiratory and cardiovascular effects associated with long-term exposure to low concentrations of volcanic gases [9,10,11]. However, evidence regarding acute exposure events remains scattered across case reports, environmental investigations, and forensic analyses. Acute intoxication events may arise from explosive eruptions, persistent soil degassing, limnic gas releases, meteorological conditions favoring gas stagnation, or accidental exposure in geothermal environments [12].

Although catastrophic events such as the Lake Nyos disaster demonstrate the devastating potential of volcanic gas emissions, numerous smaller-scale incidents have been reported worldwide [13]. These events frequently occur in the absence of eruptive activity, highlighting the importance of non-eruptive volcanic hazards.

The epidemiological characterization of such events remains incomplete due to underreporting, variability in forensic investigations, and limited environmental monitoring data. Furthermore, rescue operations in gas-rich environments pose significant risks due to rapid loss of consciousness among exposed individuals [14].

Several comprehensive reviews published in recent years have examined the health effects of volcanic gases, with particular emphasis on chronic exposure, environmental monitoring, diffuse degassing, and long-term respiratory or cardiovascular outcomes [15,16,17]. Although these studies have substantially improved our understanding of volcanic gas hazards, evidence regarding acute intoxication events and fatal human exposures remains fragmented across isolated case reports, forensic investigations, and environmental studies. To our knowledge, no systematic review has specifically synthesized the available evidence on acute accidental exposure to volcanic SO_2_, CO_2_, and H_2_S, integrating clinical, forensic, environmental, and public health perspectives. This review was therefore designed to address this knowledge gap by focusing exclusively on acute intoxication events and fatalities associated with naturally occurring volcanic gas emissions, focusing on environmental determinants, pathophysiological mechanisms and public health implications.

## 2. Materials and Methods

### 2.1. Study Design

This review was performed in accordance to the PRISMA (Preferred Reporting Items for Systematic Reviews and Meta-Analyses) guidelines [18]. This Systematic Review has not been registered on platforms such as PROSPERO. Acute exposure was effectively defined as a single or short-term exposure episode leading to symptom development or clinical outcomes during exposure or within 24 h post-exposure. The review was designed to systematically identify, critically appraise, and synthesize the available evidence regarding acute intoxication events and fatalities associated with accidental exposure to naturally occurring volcanic gases, specifically sulfur dioxide (SO_2_), carbon dioxide (CO_2_), and hydrogen sulfide (H_2_S). The review question was formulated according to the Population–Exposure–Outcome (PEO) framework (Table 1), which is considered appropriate for systematic reviews investigating the health effects of environmental and occupational exposures. The framework guided the development of the search strategy, eligibility criteria, and study selection process. Population-level ecological designs were explicitly included to capture acute community-level health surges directly linked to short-term volcanic degassing spikes, complementing individual-level forensic and clinical evidence.

Owing to the rarity of these events and the predominance of descriptive evidence, studies with heterogeneous designs, including case reports, case series, forensic investigations, ecological studies, and observational studies, were considered eligible.

### 2.2. Search Strategy and Information Sources

Six electronic databases were used to conduct a thorough literature search: PubMed/MEDLINE, Scopus, Web of Science, Embase, Google Scholar, and the World Health Organization Institutional Repository for Information Sharing (WHO IRIS). On 15 March 2026, searches were carried out that included every record that was accessible from the database’s creation to that date. Tailored keyword combinations were used to search Google Scholar, and the top 200 results were sorted by relevancy before being screened. Manual searches of official public health repositories and reference lists of relevant reviews and eligible papers were carried out independently for WHO IRIS and gray literature. No restrictions on publication year were applied. Additional manual searches of the reference lists of eligible articles and relevant reviews were performed to identify potentially eligible studies not retrieved through the electronic search. (“volcanic gas*” OR “volcanic emission*” OR fumarole* OR geothermal) AND (“carbon dioxide” OR CO2 OR “hydrogen sulfide” OR H_2_S OR “sulfur dioxide” OR SO2) AND (poisoning OR intoxication OR exposure OR fatal* OR death OR asphyxia). Equivalent search strategies were adapted for each database using the corresponding controlled vocabulary and indexing terms.

### 2.3. Eligibility Criteria

Studies were selected according to predefined eligibility criteria summarized in Table 2. Eligible studies included original investigations reporting acute human intoxication events associated with naturally occurring volcanic gases. Studies focusing exclusively on chronic exposure, non-volcanic gas poisoning, or lacking original human data were excluded.

Industrial exposures, chronic exposure studies without acute outcomes, animal experiments, and narrative reviews were excluded.

### 2.4. Study Selection

All retrieved records were exported into Rayyan where duplicates were identified and removed. Two reviewers (F.L. and D.M.) independently screened titles and abstracts according to the predefined eligibility criteria. Full-text articles considered potentially relevant were subsequently assessed independently by the same reviewers. Disagreements were resolved through discussion until consensus was reached. When consensus could not be achieved, a third reviewer was consulted (R.S.).

### 2.5. Data Extraction

Data were independently extracted by two reviewers using a standardized data extraction form. The following variables were collected:

- country;
- study design;
- volcanic setting;
- type of volcanic gas;
- exposure circumstances;
- environmental conditions;
- toxicological findings;
- clinical manifestations;
- forensic findings;
- outcomes;
- public health implications.

### 2.6. Quality Assessment

Given that most included studies consisted of case reports and case series, methodological quality was assessed using the Joanna Briggs Institute (JBI) Critical Appraisal Checklist for Case Reports and the JBI Critical Appraisal Checklist for Case Series, as appropriate [19]. Each study was independently assessed by two reviewers. Disagreements were resolved through discussion until consensus was achieved. The quality assessment was used to support interpretation of the findings rather than as a criterion for study exclusion.

### 2.7. Data Synthesis

Owing to the substantial heterogeneity in study design, exposure scenarios, reported outcomes, and available quantitative data, a meta-analysis was not considered appropriate. Therefore, findings were synthesized narratively, with particular emphasis on recurring exposure pathways, environmental determinants, toxicological mechanisms, clinical manifestations, fatal outcomes, and public health implications. Cross-study comparisons were performed to identify common patterns and evidence gaps.

## 3. Results

### 3.1. Study Selection

**Figure 1:**
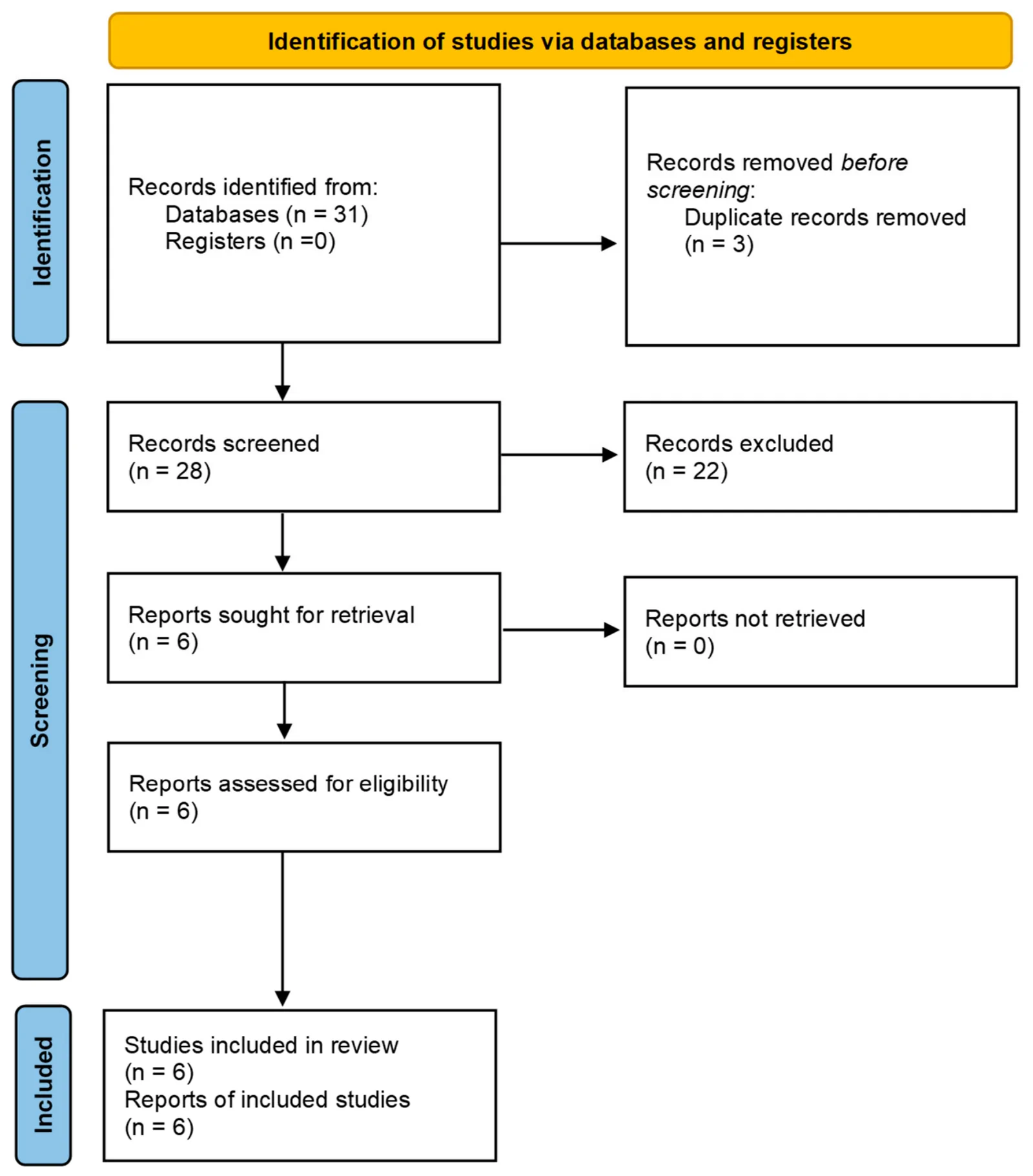
PRISMA Flow Diagram of Study Selection.

31 records in all were found in the electronic databases (PubMed/MEDLINE: n = 8; Scopus: n = 9; Web of Science: n = 6; Embase: n = 5; Google Scholar: n = 2; WHO IRIS: n = 1) as a result of the systematic search. 28 distinct records were filtered by title and abstract after three duplicates were eliminated. Twenty-two of these records were eliminated because they did not fit the eligibility requirements (such as chronic exposure emphasis, non-volcanic environments, and narrative evaluations). Six full-text reports in all were requested to be retrieved; all six were successfully retrieved and evaluated for eligibility. The six studies were included in the qualitative synthesis of the systematic review since they met all inclusion criteria [20,21,22,23,24,25]. At the full-text evaluation stage, no papers were eliminated. The PRISMA 2020 Flow Diagram (Figure 1) shows the entire screening procedure.

### 3.2. Characteristics of Included Studies

The main characteristics of the included studies are summarized in Table 3. The six studies were published between 1987 and 2022 and originated from five different countries, reflecting the global distribution of volcanic hazards. Considerable methodological heterogeneity was observed among the included publications, comprising case reports, forensic investigations, an ecological time-series study, and a large-scale disaster investigation.

The studies investigated acute exposure to the principal toxic volcanic gases, including carbon dioxide (CO_2_), hydrogen sulfide (H_2_S), and sulfur dioxide (SO_2_), either individually or in combination. Exposure occurred in a variety of volcanic settings, such as geothermal fields, fumaroles, volcanic craters, diffuse degassing areas, and limnic volcanic lakes, highlighting the diversity of hazardous environments capable of generating life-threatening gas concentrations.

Despite differences in study design and geographic setting, all included studies focused on acute intoxication events, describing clinical manifestations ranging from mild respiratory symptoms to rapid cardiorespiratory collapse and death. Several investigations incorporated environmental monitoring, forensic examinations, or toxicological analyses to support exposure assessment and the attribution of health events to environmental exposure rather than simple individual susceptibility.

### 3.3. Methodological Quality Assessment

Supplementary Table S2 displays the comprehensive item-level JBI critical assessments for each of the included investigations. Forensic investigations, observational designs, and descriptive case reports make up the majority of the evidence base. Due to their retrospective design, small sample sizes, and incapacity to determine population-level risk thresholds, case reports [21,22,23,24] generally showed excellent internal validity with regard to clinical presentation, postmortem results, and descriptions of the environmental background. Although individual exposure quantification was limited by emergency settings, the Lake Nyos disaster study [25] produced strong multidisciplinary evidence integrating clinical, environmental and geological data.

Despite using sophisticated statistical modeling (generalized additive models controlling for seasonality, meteorological conditions, and epidemic patterns), the ecological time-series study [20] is still vulnerable to the intrinsic drawbacks of ecological designs, including the possibility of ecological fallacy. The data was assessed qualitatively as offering reliable attribution for particular acute exposure events but emphasizing the necessity of cautious extrapolation to larger populations, as opposed to classifying research with subjective quality scores (Table 4).

Most case reports and forensic investigations provided detailed descriptions of clinical presentation, environmental circumstances, toxicological findings, and patient outcomes, thereby supporting the validity of exposure attribution. However, their retrospective nature, limited sample size, and lack of control groups restricted external validity and prevented causal inference beyond the reported events.

The ecological study contributed valuable population-level information regarding the association between volcanic gas exposure and health outcomes but was subject to the limitations typical of ecological analyses, including potential ecological fallacy and the inability to determine individual exposure. Nevertheless, no study presented methodological limitations severe enough to justify exclusion from the qualitative synthesis.

### 3.4. Synthesis of Evidence

#### 3.4.1. Exposure Settings and Toxic Gases

Across the included studies, hazardous exposure occurred in a wide range of volcanic environments characterized by active gas emissions. The most frequently reported settings included fumarolic fields, geothermal areas, volcanic craters, diffuse degassing zones, and volcanic lakes, where volcanic gases may accumulate to dangerous concentrations, particularly under conditions of limited atmospheric dispersion.

Carbon dioxide (CO_2_) was the gas most frequently associated with fatal incidents because of its tendency to accumulate in low-lying or poorly ventilated areas, causing oxygen displacement and rapid asphyxia. Hydrogen sulfide (H_2_S) was also commonly implicated in severe intoxication events owing to its high acute toxicity and inhibition of cellular respiration, whereas sulfur dioxide (SO_2_) was primarily associated with acute respiratory irritation and pulmonary injury following exposure to elevated concentrations. Several studies described simultaneous exposure to multiple gases, emphasizing the complex toxicological environment typical of active volcanic systems.

#### 3.4.2. Clinical Manifestations and Fatal Outcomes

Clinical manifestations varied according to gas composition, concentration, and duration of exposure but showed several recurring patterns across the included studies. Frequently reported symptoms included dyspnea, cough, dizziness, headache, nausea, loss of consciousness, and acute respiratory distress. In severe cases, exposure rapidly progressed to pulmonary edema, respiratory failure, cardiovascular collapse, and sudden death.

Fatal outcomes were predominantly attributed to oxygen displacement by carbon dioxide, resulting in hypoxia and asphyxia, or to the direct toxic effects of hydrogen sulfide on mitochondrial respiration. Forensic investigations consistently demonstrated that high environmental gas concentrations, together with the absence of adequate warning systems or delayed rescue, substantially increased mortality risk.

#### 3.4.3. Environmental Determinants of Exposure

Several environmental factors consistently emerged as contributors to hazardous exposure scenarios (Table 5). Gas accumulation was frequently observed in topographic depressions, enclosed spaces, volcanic craters, caves, and areas characterized by limited air circulation, where dense gases such as carbon dioxide can persist close to ground level. Meteorological conditions, including low wind speed, atmospheric stability, and temperature inversion, further favored the accumulation of toxic gases and increased the likelihood of accidental exposure.

In addition, sudden increases in volcanic degassing or limnic gas release were identified as major determinants of mass casualty events, demonstrating that both chronic diffuse emissions and abrupt geological phenomena may represent significant public health hazards.

#### 3.4.4. Public Health Implications

Although the included studies primarily focused on individual intoxication events, several common public health implications emerged from the available evidence. Continuous environmental monitoring, early warning systems, hazard mapping, visitor education, restricted access to high-risk areas, and the use of portable gas detection devices were consistently identified as key preventive strategies for reducing morbidity and mortality associated with volcanic gas exposure.

The evidence further highlighted the importance of multidisciplinary collaboration among volcanologists, environmental scientists, emergency responders, occupational health professionals, and public health authorities to improve risk communication, emergency preparedness, and surveillance in volcanically active regions. Despite the limited number of available studies, the consistency of these recommendations across diverse geographic settings underscores their relevance for future prevention strategies.

## 4. Discussion

### 4.1. Principal Findings

**Figure 2:**
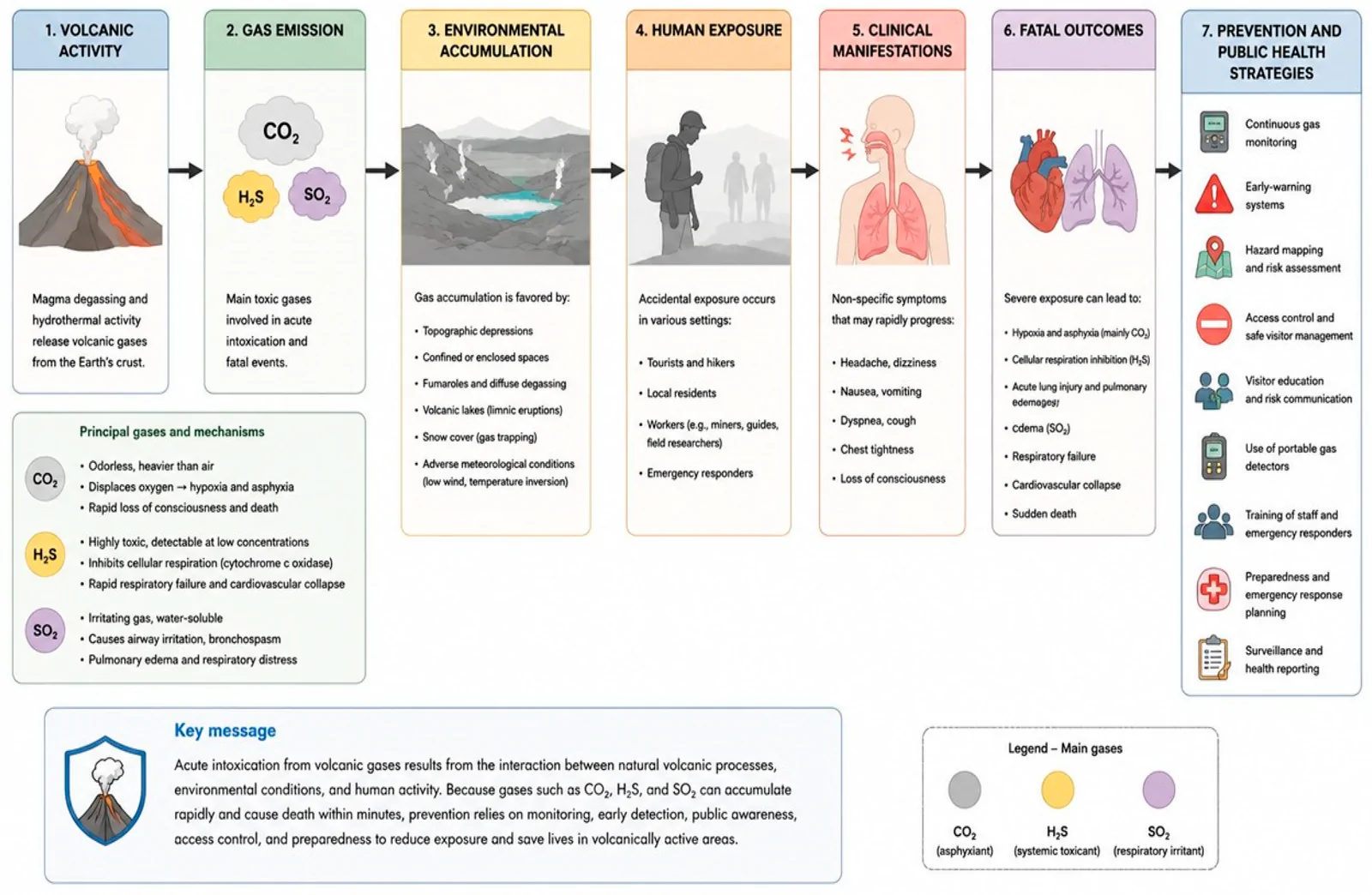
Schematic overview of the mechanisms, health effects, and prevention strategies associated with acute volcanic gas exposure.

The events analyzed in this review consistently demonstrate that exposure to carbon dioxide (CO_2_) and hydrogen sulfide (H_2_S) may lead to rapid loss of consciousness through distinct yet equally hazardous mechanisms (Figure 2). Carbon dioxide primarily acts as an asphyxiant by displacing oxygen in the surrounding air. At concentrations exceeding 20–30%, this oxygen displacement produces severe hypoxia and explains the rapid collapse observed in enclosed cavities, fumaroles, and topographically depressed environments [26].

In contrast, hydrogen sulfide exerts a direct toxic effect at the cellular level by inhibiting cytochrome oxidase activity, thereby disrupting mitochondrial respiration and inducing histotoxic hypoxia [27]. At concentrations above 500–1000 ppm, such as those measured in the Solfatara of Pozzuoli incident, H_2_S can produce the so-called knockdown effect, characterized by almost immediate loss of consciousness [28].

Environmental accumulation of volcanic gases emerges as a crucial determinant of acute exposure events. Gas buildup in topographic depressions, poorly ventilated areas, or locations covered by snow significantly increases exposure risk [29]. The incidents reported in California and Italy demonstrate how relatively minor meteorological variations, including low wind speed, temperature inversion, or snow cover, may transform apparently safe environments into highly hazardous settings. Furthermore, the cases observed in Réunion Island illustrate that nocturnal gas stagnation may occur even in open-air environments, indicating that confined spaces are not a prerequisite for lethal exposure. Although the included studies originated from different geographical settings and adopted heterogeneous methodologies, they consistently highlighted the importance of environmental conditions, gas accumulation, and delayed recognition of exposure in determining clinical severity and mortality. An important finding of this review is that severe intoxication events rarely result from a single factor. Instead, adverse outcomes generally arise from the interaction between geological activity, environmental conditions, human behaviour, and the absence of effective preventive measures. This multifactorial nature emphasizes that volcanic gas intoxication should not be considered solely a toxicological problem but also a public health challenge requiring integrated environmental surveillance, risk communication, and emergency preparedness.

### 4.2. Implications for Safety in Volcanic Areas and Visitor Protection

A recurrent feature across the analyzed cases is the vulnerability of tourists and occasional visitors, who are often unaware of the risks associated with natural volcanic degassing. Hidden cavities, fumaroles concealed by snow, and inadequately marked geothermal areas represent major contributing factors in the reported fatalities. The Solfatara accident, which involved an entire family, and the fatal incident involving geology students in Réunion Island both highlight the urgent need for more effective safety measures in volcanic and geothermal areas [30].

Preventive strategies should include the restriction and control of access to high-risk geothermal zones, the installation of permanent gas monitoring systems, temporary closures during adverse meteorological conditions, and improved training for local personnel and visitors regarding specific environmental hazards. Given the rapid onset of fatal events associated with high gas concentrations, effective risk communication and evidence-based preventive behavior are essential components of public safety management in volcanic regions.

### 4.3. Population-Level Health Impact

In contrast to individual fatal events, only one of the included studies assessed the health impact of volcanic gas exposure at the population level. Non-eruptive volcanic degassing can result in significant health costs for the entire society, according to population-level epidemiological data from the Democratic Republic of the Congo [20]. Increased daily outpatient visits for acute respiratory complaints were substantially correlated with elevated tropospheric gases (IRR = 1.08, 95% CI: 1.02–1.14). However, the absence of a consistent temporal association between eruptions and respiratory outcomes may reflect confounding factors, including humanitarian crises, population displacement, and other environmental influences. These findings underscore the need for more robust epidemiological investigations to better quantify the long-term health impact of volcanic gas exposure.

The catastrophic Lake Nyos event represents a unique example of large-scale volcanic gas exposure and confirms that limnic degassing processes can produce devastating consequences, resulting in thousands of deaths within a short period. Understanding limnological dynamics and monitoring lake stratification stability remain essential for preventing similar events in other volcanic lake systems.

### 4.4. Underestimation of Cases and Gaps in the Literature

Despite the well-recognized presence of volcanic gas hazards in many regions worldwide, the limited number of studies identified in this review suggests that acute exposure events may be substantially underreported. Several important gaps in the literature were identified.

First, there is a lack of standardized surveillance systems for acute health events related to volcanic gas exposure. Second, substantial variability exists in forensic protocols and toxicological investigations, limiting comparability across cases. Third, non-fatal incidents are rarely documented in the scientific literature, despite their potential relevance for risk assessment. Finally, simultaneous environmental measurements during exposure events are often unavailable, hindering the identification of critical gas concentration thresholds associated with adverse outcomes.

These limitations are particularly relevant in countries characterized by intense volcanic activity but limited resources for environmental monitoring and systematic health data collection.

## 5. Strengths and Limitations of the Review

A major strength of this review is the comprehensive search strategy, which included multiple databases and allowed the identification of diverse events across heterogeneous geographical contexts. The integration of clinical, environmental, and forensic evidence provides a multidisciplinary perspective on acute volcanic gas exposure.

Nevertheless, several limitations should be acknowledged. The number of available studies was relatively small, quantitative data were not comparable across studies, and the heterogeneity of case reports precluded meta-analysis. Additionally, variability in methodological quality among included studies may have influenced the findings. Despite these limitations, the review provides a useful framework for guiding future research and informing risk prevention strategies.

## 6. Conclusions

This review demonstrates that acute exposure to volcanic gases can lead to sudden and fatal events across a wide range of environmental contexts. Rapid loss of consciousness followed by pulmonary edema and asphyxia represents a recurrent clinical pattern documented across several fatal events Although such events are relatively rare, they are often underestimated and difficult to prevent without adequate environmental monitoring and effective risk communication.

Overall, a multidisciplinary framework integrating environmental monitoring, toxicological assessment, epidemiological surveillance and research on individual susceptibility to exposures is essential to strengthen public health protection in volcanically active regions [31,32,33,34,35,36].

The available evidence supports the need to improve early warning and detection systems in volcanic areas, regulate tourist access to active degassing zones, enhance training for responders regarding the knockdown effect, and integrate health, geological, and meteorological data to optimize preventive strategies [37,38].

### Future Research Priorities and Risk Governance

The available evidence highlights several priorities for future research and public health intervention. Continuous monitoring networks for volcanic gas emissions should be implemented in volcanic and geothermal areas to enable early detection of hazardous conditions. Integration of geological, meteorological, and health data could support predictive models for risk assessment. Improved training and safety protocols for tourism operators, emergency responders, and local populations are also necessary. Furthermore, the development of international guidelines for the management of non-eruptive volcanic gas hazards and the promotion of longitudinal epidemiological studies would improve understanding of the long-term health effects of intermittent or persistent exposure.

## Figures and Tables

**Table 1:** Research question according to the PEO framework.

	Description
Population (P)	Individuals of any age who are exposed to naturally occurring volcanic gasses include visitors, locals, emergency personnel, and employes who operate in volcanic regions (such as park rangers, volcano guides, field geoscientists and researchers).
Exposure (E)	Acute accidental exposure to volcanic sulfur dioxide (SO_2_), carbon dioxide (CO_2_), and/or hydrogen sulfide (H_2_S) in volcanic, geothermal, fumarolic, or limnic environments.
Outcome (O)	Acute intoxication, poisoning, respiratory compromise, neurological manifestations, pulmonary edema, asphyxia, sudden death, forensic findings, and other clinically documented health outcomes associated with volcanic gas exposure.

**Table 2:** Eligibility criteria for study inclusion and exclusion.

Domain	Inclusion Criteria	Exclusion Criteria
Population	Human subjects of any age or sex	Animal studies, in vitro studies
Exposure	Acute accidental exposure to naturally occurring volcanic SO_2_, CO_2_ and/or H_2_S	Occupational exposures that are either commercial, industrial, or non-volcanic; artificial gas leaks
Setting	Volcanic environments, geothermal fields, fumaroles, volcanic craters, limnic lakes, diffuse degassing areas	Non-volcanic environmental settings
Outcomes	Acute intoxication, poisoning, respiratory impairment, neurological manifestations, pulmonary edema, asphyxia, sudden death, forensic findings	Chronic health effects only, environmental monitoring without human health outcomes
Study design	Case reports, case series, forensic investigations, observational studies, ecological studies reporting original data	Reviews, systematic reviews, meta-analyses, editorials, letters without original data, conference abstracts
Publication characteristics	Peer-reviewed articles published in English or Italian	Publications in other languages or without accessible full text

**Table 3:** Summary of Health Events Associated with Exposure to Volcanic Gases.

Evidence Tier	Study	Country/Setting	Study Design	Gas	Exposure Circumstances	Main Health Outcomes	Environmental/Toxicological Evidence
Population-level	Michellier et al. [19]	DR Congo	Ecological time-series	SO_2_/volcanic emissions	Volcanic degassing	Acute respiratory morbidity	Environmental/remote-sensing exposure assessment
Individual forensic	Carfora et al. [20]	Solfatara, Italy	Forensic case report	CO_2_, H_2_S	Soil/fumarolic degassing	Fatal asphyxia, pulmonary edema	Autopsy/toxicological and environmental evidence
Individual forensic	Pefferkorn et al. [21]	Réunion Island	Forensic case report	H_2_S, SO_2_	Peri-volcanic exposure/gas stagnation	Fatal pulmonary injury	Forensic and environmental reconstruction
Individual clinical/forensic	Cantrell & Young [22]	California, USA	Case report	H_2_S, SO_2_, CO_2_	Fumarole exposure	Fatal outcome	Clinical/environmental reconstruction
Individual clinical	Hill [23]	California, USA	Case report	CO_2_	Volcanic degassing	Asphyxia/pulmonary edema	Environmental evidence
Disaster-level	Kling et al. [24]	Lake Nyos, Cameroon	Disaster investigation	CO_2_	Limnic gas release	Mass asphyxiation	Multidisciplinary geological/environmental evidence

**Table 4:** Methodological quality assessment of the included studies according to the Joanna Briggs Institute (JBI) Critical Appraisal Tools.

Study	Study Design	JBI Appraisal Tool	Main Strengths	Main Limitations/Potential Bias
Kling et al., 1987 [24]	Disaster investigation	JBI Analytical Cross-Sectional Studies	Large-scale multidisciplinary investigation; environmental and clinical evidence	Retrospective design; limited individual clinical data
Hill, 2000 [23]	Case report	JBI Case Reports	Detailed clinical and environmental description	Single patient; limited generalizability
Cantrell & Young, 2009 [22]	Case report	JBI Case Reports	Well-documented exposure and clinical evolution	Retrospective design; absence of comparison group
Carfora et al., 2019 [20]	Forensic case report	JBI Case Reports	Comprehensive autopsy and toxicological investigation	Single fatal event
Michellier et al., 2020 [19]	Ecological time-series study	JBI Analytical Cross-Sectional Studies	Population-based environmental assessment; statistical analysis	Ecological fallacy; no individual exposure assessment
Pefferkorn et al., 2022 [21]	Forensic case report	JBI Case Reports	Detailed forensic reconstruction; toxicological evidence	Small sample size; retrospective design

**Table 5:** Main Characteristics of Acute Volcanic Gas Exposure.

Variable	Findings
Main gases	CO_2_, H_2_S, SO_2_
Exposure environments	Cavities, fumaroles, geothermal areas
Triggering conditions	Gas accumulation, meteorological stagnation
Clinical presentation	Rapid unconsciousness, pulmonary edema
Forensic findings	Cyanosis, cerebral microhemorrhages
Risk groups	Tourists, workers, rescuers

## Data Availability

The original contributions presented in this study are included in the article and Supplementary Materials. Further inquiries can be directed to the corresponding authors.

## Supplementary Materials

The following supporting information can be downloaded at: https://www.mdpi.com/article/10.3390/medsci14050531/s1.
