# Supplementary material for: Acute Events and Fatal Outcomes Following Exposure to Volcanic Gases (SO2, CO2, H2S): A Systematic Review
Source: Med Sci (Basel). 2026 Aug 31;14(5):531. doi: 10.3390/medsci14050531 (PMC13609723; doi:10.3390/medsci14050531)
Supplement: Supplementary file 1 [file medsci-14-00531-s001.zip › medsci-4441795-supplementary.pdf]

Supplementary Table S1. Full Database-Specific Search Strategies

Database	Search Query & Syntax Adaptations	Filters / Limits Applied	Notes / Screening Protocol
PubMed / MEDLINE	(("Volcanic Gases"[Mesh] OR "Volcanoes"[Mesh] OR "volcanic gas*"[Title/Abstract] OR "volcanic emission*"[Title/Abstract] OR fumarole*[Title/Abstract] OR geothermal[Title/Abstract]) AND ("Carbon Dioxide"[Mesh] OR "Hydrogen Sulfide"[Mesh] OR "Sulfur Dioxide"[Mesh] OR "carbon dioxide"[Title/Abstract] OR CO2[Title/Abstract] OR "hydrogen sulfide"[Title/Abstract] OR H2S[Title/Abstract] OR "sulfur dioxide"[Title/Abstract] OR SO2[Title/Abstract]) AND ("Poisoning"[Mesh] OR "Asphyxia"[Mesh] OR poisoning[Title/Abstract] OR intoxication[Title/Abstract] OR exposure[Title/Abstract] OR fatal*[Title/Abstract] OR death[Title/Abstract] OR asphyxia[Title/Abstract]))	None applied. Search cutoff date: March 15, 2026.	Standard MeSH index terms combined with title/abstract free-text keywords.
Scopus	TITLE-ABS-KEY(("volcanic gas*" OR "volcanic emission*" OR fumarole* OR geothermal) AND ("carbon dioxide" OR CO2 OR "hydrogen sulfide" OR H2S OR "sulfur dioxide" OR SO2) AND (poisoning OR intoxication OR exposure OR fatal* OR death OR asphyxia))	Document Types: Article, Review, Short Survey, Note. Cutoff date: March 15, 2026.	Title, Abstract, and Keyword fields searched using wildcards (*).
Web of Science *(Core Collection)*	TS=(("volcanic gas*" OR "volcanic emission*" OR fumarole* OR geothermal) AND ("carbon dioxide" OR CO2 OR "hydrogen sulfide" OR H2S OR "sulfur dioxide" OR SO2) AND (poisoning OR intoxication OR exposure OR fatal* OR death OR asphyxia))	None applied. Search cutoff date: March 15, 2026.	Topic search (TS) covering Title, Abstract, Author Keywords, and Keywords Plus.
Embase *(Elsevier)*	('volcanic gas'/exp OR 'volcano'/exp OR 'volcanic gas*':ti,ab OR 'volcanic emission*':ti,ab OR 'fumarole*':ti,ab OR 'geothermal':ti,ab) AND ('carbon dioxide'/exp OR 'hydrogen sulfide'/exp OR 'sulfur dioxide'/exp OR 'carbon dioxide':ti,ab OR 'co2':ti,ab OR 'hydrogen sulfide':ti,ab OR 'h2s':ti,ab OR 'sulfur dioxide':ti,ab OR 'so2':ti,ab) AND ('intoxication'/exp OR 'asphyxia'/exp OR 'poisoning':ti,ab OR 'intoxication':ti,ab OR 'exposure':ti,ab OR 'fatal*':ti,ab OR 'death':ti,ab OR 'asphyxia':ti,ab)	None applied. Search cutoff date: March 15, 2026.	Emtree exploded terms (/exp) combined with title/abstract (:ti,ab) text searching.
Google Scholar	allintitle: volcanic OR geothermal OR fumarole "carbon dioxide" OR "hydrogen sulfide" OR "sulfur dioxide" fatal OR asphyxia OR intoxication	Sorted by relevance. Search cutoff	First 200 records screened based on relevance sorting

		date: March 15, 2026.	to identify grey literature.
WHO IRIS *(Repository)*	volcanic gas AND (poisoning OR asphyxia OR death)	None applied. Search cutoff date: March 15, 2026.	Manual screening of official institutional reports and disaster technical documentation.

Supplementary Table S2. Item-Level Critical Appraisal Results Using JBI Tools

Part A: JBI Critical Appraisal Checklist for Case Reports

Checklist Criteria:

Q1: Patient demographics clearly described? | **Q2:** Patient history clearly described? | **Q3:** Current clinical condition clearly described? | **Q4:** Diagnostic tests/assessment results clearly described? | **Q5:** Intervention/treatment procedure clearly described? | **Q6:** Post-intervention clinical condition clearly described? | **Q7:** Adverse events/unanticipated events identified? | **Q8:** Take-away lessons provided?

Study	Q1	Q2	Q3	Q4	Q5	Q6	Q7	Q8	Overall Methodological Assessment
Carfora et al. (2019)	Yes	Yes	Yes	Yes	N/A	N/A	Yes	Yes	Low risk of bias: Highly detailed autopsy and toxicological investigation.
Pefferkorn et al. (2022)	Yes	Yes	Yes	Yes	N/A	N/A	Yes	Yes	Low risk of bias: Comprehensive forensic reconstruction and lung histology.
Cantrell & Young (2009)	Yes	Yes	Yes	Yes	N/A	N/A	Yes	Yes	Low risk of bias: Clear exposure context and post-mortem evaluation.
Hill (2000)	Yes	Yes	Yes	Yes	N/A	N/A	Yes	Yes	Low risk of bias: Detailed clinical presentation and environmental description.

Note: Items Q5 and Q6 were marked as "N/A" (Not Applicable) for fatal exposure events where no medical interventions or post-treatment follow-ups were possible.

Part B: JBI Critical Appraisal Checklist for Analytical Cross-Sectional / Observational Studies (Adapted)

Checklist Criteria:

Q1: Inclusion criteria clearly defined? | **Q2:** Study subjects and setting described in detail? | **Q3:** Exposure measured in a valid and reliable way? | **Q4:** Objective, standard criteria used for condition measurement? | **Q5:** Confounding factors identified? | **Q6:** Strategies to deal with confounders stated? | **Q7:** Outcomes measured in a valid and reliable way? | **Q8:** Appropriate statistical analysis used?

Study	Q1	Q2	Q3	Q4	Q5	Q6	Q7	Q8	Overall Methodological Assessment
Kling et al. (1987) *(Disaster Investigation)*	Yes	Yes	Unclear	Yes	Unclear	No	Yes	N/A	High descriptive value: Retrospective disaster assessment; individual exposure metrics unmeasurable.

Michellier et al. (2020) *(Ecological Time-Series)*	Yes	Yes	Yes		Yes	Yes		Yes	Yes	Yes	Methodologically robust: GAM modeling controlling for seasonality, meteorology, and lag effects.
